# Melatonin-loaded lipid-core nanocapsules protect against lipid peroxidation caused by paraquat through increased SOD expression in *Caenorhabditis elegans*

**DOI:** 10.1186/s40360-019-0352-4

**Published:** 2019-12-19

**Authors:** Mariele F. Charão, Gabriela Goethel, Natália Brucker, Karina Paese, Vera L. Eifler-Lima, Adriana R. Pohlmann, Silvia S. Guterres, Solange C. Garcia

**Affiliations:** 1 Laboratory of Toxicology (LATOX), Federal University of Rio Grande do Sul, Porto Alegre, Brazil, Porto Alegre, Rio Grande do Sul Brazil; 2 Laboratory of Analytical Toxicology, Feevale University, Novo Hamburgo, Brazil, Novo Hamburgo, RS Brazil; 3 Post-graduate Program in Pharmaceutical Sciences, Federal University of Rio Grande do Sul, Porto Alegre, Brazil, Porto Alegre, RS Brazil; 4 Department of Physiology and Pharmacology, Federal University of Santa Maria, Porto Alegre, Brazil, Santa Maria, RS Brazil

**Keywords:** Nanocapsules, Antioxidant, *C. elegans*, Herbicide, Superoxide dismutase

## Abstract

**Background:**

Melatonin has been described in the literature as a potent antioxidant. However, melatonin presents variable, low bioavailability and a short half-life. The use of polymeric nanoparticulated systems has been proposed for controlled release. Thus, the purpose of this study was to investigate the action of melatonin-loaded lipid-core nanocapsules (Mel-LNC) in the antioxidant system of *Caenorhabditis elegans,* and the possible protective effect of this formulation against lipid peroxidation caused by paraquat (PQ).

**Methods:**

The suspensions were prepared by interfacial deposition of the polymer and were physiochemically characterized. *C. elegans* N2 wild type and transgenic worm CF1553, muls84 [sod-3p::gfp; rol6(su1006)] were obtained from the Caenorhabditis Genetics Center (CGC). The worms were divided into 5 groups: Control, PQ 0.5 mM, PQ 0.5 mM + Mel-LNC 10 μg/mL, PQ + unloaded lipid-core nanocapsules (LNC), and PQ + free melatonin (Mel) 10 μg/mL. The lipid peroxidation was assessed through thiobarbituric acid (TBARS) levels and the fluorescence levels of the transgenic worms expressing GFP were measured.

**Results:**

The LNC and Mel-LNC presented a bluish-white liquid, with pH values of 5.56 and 5.69, respectively. The zeta potential was − 6.4 ± 0.6 and − 5.2 ± 0.2, respectively. The mean particle diameter was 205 ± 4 nm and 203 ± 3 nm, respectively. The total melatonin content was 0.967 mg/ml. The TBARS levels were significantly higher in the PQ group when compared to the control group (*p* < 0.001). Mel-LNC reduced TBARS levels to similar levels found in the control group. Moreover, only Mel-LNC significantly enhanced the SOD-3 expression (*p* < 0.05). Mel-LNC was capable of protecting *C. elegans* from lipid peroxidation caused by PQ and this was not observed when free melatonin was used. Moreover, Mel-LNC increased the fluorescence intensity of the transgenic strain that encodes the antioxidant enzyme SOD-3, demonstrating a possible mechanism of protection from PQ-induced damage.

**Conclusion:**

These findings demonstrated that melatonin, when associated with nanocapsules, had improved antioxidant properties and the protective activity against PQ-induced lipid peroxidation could be associated with the activation of antioxidant enzymes by Mel-LNC in *C. elegans*.

## Background

Melatonin, a hormone produced by the pineal glandule, has a high number of effects that are described in the literature, such as sedative, analgesic, and anticarcinogenic effects [1]. Also, it is reported that melatonin acts as a potent antioxidant and a reactive oxygen species (ROS) scavenger [2]. In addition, it has been observed that melatonin stimulates the activation of antioxidant enzymes, inhibiting pro-oxidative enzymes [3], and enhances the efficacy of mitochondrial functions [4, 5], regulating a large number of molecular pathways, including oxidative stress, inflammation, apoptosis, and cell death in different contexts [2–5]. However, melatonin presents a short half-life, variable and low bioavailability, and is quickly metabolized by the liver and is therefore not the best option for conventional formulations.

Several studies on nanotechnology have shown that this technology has a promising innovative drug release system, improving the properties of some molecules [6–9]. An improvement of the biological action of drugs has been demonstrated when associated with polymeric nanocapsules prepared with a biodegradable and biocompatible polymer, poly(ɛ-caprolactone) (PCL) [7]. Controlled systems delivery using polymeric nanoparticulated systems is being used to improve the properties of melatonin [8]. Schaffazick et al. [9] demonstrated that the encapsulation of melatonin, in polymeric nanocapsules, improved the antioxidant properties of this molecule as it caused a reduction in lipid peroxidation in the brain and liver of *Wistar* rats. Recently, our research group reported that melatonin-loaded lipid-core nanocapsules (Mel-LNC), prepared with PCL, presented protective effects against cytotoxicity and genotoxicity caused by paraquat (PQ) in the A549 cell line [10] and reduced ROS production in *Caenorhabditis elegans* exposed to PQ [11].

Paraquat (PQ) is an herbicide used in agriculture and it is highly toxic for humans and animals, being responsible for many cases of acute poisoning and death [12]. The main mechanism of toxicity of PQ is the generation of reactive oxygen species, leading to oxidative stress (OS). This process could result in deleterious effects such as lipid peroxidation, protein damage, genotoxicity, and NADPH oxidation, leading to the disruption of biochemical processes where NADPH is required [13–17].

Alternative models are a useful tool used in pharmacology and toxicological evaluations during initial studies. *C. elegans* is one of the best-established animal models and a very attractive experimental model due to its various characteristics: small size, short lifespan, rapid life cycle, translucent body, ability to self-fertilize and high reproductive rate, low cost, easiness to handle, and high degree of shared orthology with the human genome [18, 19]. Moreover, it is possible to verify the possible mechanism of action through green fluorescent protein (GFP) labelled strains [20]. However, there is still a lack of studies concerning the possible mechanism of action of Mel-LNC. Therefore, this study aimed to investigate the activity of Mel-LNC in the antioxidant system in an alternative in vivo model of *Caenorhabditis elegans,* and the possible protective effect of this formulation against lipid peroxidation caused by PQ.

## Methods

### Chemicals

Sorbitan monostearate and melatonin were obtained from Sigma-Aldrich (Strasbourg, France). Biodegradable polymer poly(ɛ-caprolactone) (PCL) (MW = 50,000) was supplied by Capa (Toledo, Ohio, USA). Caprylic/capric triglyceride and polysorbate 80 were obtained from Delaware (Porto Alegre, Brazil). Bacto-agar and bacto-peptone were obtained from Becton Dickinson BD® (New Jersey, USA) and HiMedia Laboratories® (Mumbai, India). Phosphoric acid and 2-thiobarbituric acid were purchased from Tedia Co (Fairfield, Ohio, USA) and Sprectrum Chemical Co (Gardena, California, USA), respectively. All other chemicals and solvents were analytical or pharmaceutical grade.

### Preparation and physicochemical characterization of the lipid-core nanocapsules

The suspensions were prepared by interfacial deposition of the polymer [21, 22]. The organic phase was constituted of melatonin, sorbitan monostearate, capylic/capric triglyceride, and PCL, and then acetone was added in the aqueous phase, which was constituted of water and polysorbate 80, under agitation. After that, the organic solvent and a fraction of the water were evaporated, and a white opaque liquid product was obtained. The final volume was adjusted to a theoretical final melatonin content of 1 mg/mL. The blank formulation of unloaded lipid-core nanocapsules (LNC) was prepared as according to previously described without the addition of melatonin.

Particle size and distribution analyses were performed by dynamic light scattering using backscatter detection at 173° (Zetasizer ZS, Malvern, UK) and volume-weighted mean diameter ([D4,3]) was determined by laser diffractometry at 25 °C (Mastersizer 2000, Malvern, UK). A calibrated potentiometer (FiveEasy, Mettler Toledo, Brazil) was used to measure the pH values at 25 °C. The zeta potential was determined by electrophoretic light scattering (ZetasizerNano ZS model ZEN 3600, Malvern, USA).

The total amount of melatonin in the formulation was determined by a high-performance liquid chromatograph equipped with an ultraviolet-visible detector (Elmer Series 200 chromatograph), as according to Schaffazick et al. [9].

Encapsulation efficiency (EE%) was determined indirectly by measuring free drug concentration (C_f_), according to the equation: EE% = (C_f_ - *C*_*total*_)/ *C*_*total*_ × 100, where *C*_*total*_ is the total concentration of melatonin in the formulation. The formulation (Mel-LNC) was put into an ultrafiltration-centrifugation unit (Millipore, Amicon® Ultra, cutoff 10,000 Da), centrifuging (10 min, 15,300×g) and injecting the ultrafiltrate in the HPLC system as according to Schaffazick et al. [9].

### Strains

The *C. elegans* N2 (wild-type) strains and the green fluorescent protein (GFP)-marked strain CF1553 [muls84] superoxide dismutase-3 (SOD::GFP) were maintained on nematode growth medium (NGM) plates seeded with *Escherichia coli* OP50 at 20 °C.

### Synchronization

Gravid *C. elegans* were rinsed off the plates into centrifuge tubes and were lysed with a bleaching mixture (1% NaOCl; 0.25 M NaOH), followed by flotation in a 30% sucrose solution (m/v) to separate the eggs. The eggs were washed with M9 buffer (0.02 M KH_2_PO_4_, 0.04 M Na_2_HPO_4_, 0.08 M NaCl, and 0.001 M MgSO_4_) and allowed to hatch overnight on NGM agar plates without bacteria [23].

### Treatment

Without using bacteria, 2500 previously synchronized L1 worms were divided into 5 groups: Control, PQ 0.5 mM, PQ 0.5 mM + Mel-LNC 10 μg/mL, PQ 0.5 mM + LNC, and PQ 0.5 mM + free melatonin (Mel) 10 μg/mL in 0.5% NaCl liquid media. The PQ + Mel-LNC, PQ + LNC, and PQ + Mel groups were pre-treated for 30 min at 20 °C, by constant agitation in a rotator, with Mel-LNC, LNC, or Mel. After three washes with 0.5% NaCl, the worms were exposed to PQ 0.5 mM for 30 min at 20 °C, by constant agitation. The PQ concentration chosen was based on a previous study [11], where 30% worm mortality and ROS enhancement was observed, at a concentration of PQ 0.5 mM, compared to the control group. The Mel-LNC and Mel concentrations were based on a previous study by our research group [11]. Additionally, worms treated with saline were used as a control. After PQ exposure, the worms were washed 3 times with 0.5% NaCl to remove the treatments and then transferred to NGM recovery plates inoculated with *Escherichia coli* - OP50 for posterior assays.

### Thiobarbituric acid (TBARS) assay

Thiobarbituric acid reactive substances (TBARS) were determined in the adult worms, 48 h after the end of treatment, as a marker of lipid peroxidation for the TBA (thiobarbituric acid) assay using a 1,1,3,3-tetramethoxypropane solution as malondialdehyde (MDA) standard [24]. The plates containing the worms were washed to remove the OP50. The nematodes were sonicated in turrax homogenizer at full amplitude for about 60 s, in order to release the lipid and protein content. Then the content was centrifuged at 12,000 g for 5 min. The supernatant was transferred to cryotubes where the TBARS reaction happened, with the addition of 0.1 M phosphoric acid solution, 20 mM sodium dodecyl sulfate solution, and 40 mM 2-thiobarbituric acid solution. The reaction took place in a water bath for 1 h and 30 min under agitation at 100 °C. Additionally, the samples were transferred to 96 well plates and their absorbance was read at 532 nm (Spectramax Me2; Molecular Devices LLC, Sunnyvale, CA, USA). The protein content of the samples was determined as according to Bradford [25].

### SOD fluorescence quantification

The GFP expressing strain (CF1553 [muls84]) were subjected to acute exposure as described above. One thousand five hundred L1 worms were maintained in 100 μL of saline buffer and transferred to a 96 well plate, where total GFP fluorescence was measured after 1 h of treatment using 485 nm excitation and 530 nm emission filters and a microplate reader (Spectramax Me2, Molecular DevicesLLC, Sunnyvale, CA, USA) at 20 °C. The results were expressed as the mean percentage of fluorescence intensity relative to control wells.

### Statistical analysis

The results were expressed as the mean ± standard deviation (SD). All figures were generated using GraphPad Prism (GraphPad Software, Inc.). The normality of data was tested by the Shapiro-Wilk test. The statistical analysis of significance was carried out using ANOVA, followed by the post hoc Bonferroni test in the SPSS Statistics software (version 22). Values of *p* ≤ 0.05 were considered statistically significant.

## Results

### Preparation and characterization of LNC

Both formulations, LNC and Mel-LNC, presented a bluish-white liquid macroscopic aspect, with pH values ranging from 5.56 to 5.69. Particle sizing analysis by dynamic light scattering showed hydrodynamic diameters (Z-average) ranging from 200 to 209 nm (Table 1). The LNC and Mel-LNC presented low polydispersity indexes and a narrow distribution (Table 1). The zeta potential for the LNC and Mel-LNC was − 6.4 ± 0.6 and − 5.2 ± 0.2, respectively. The total melatonin content was 0.967 mg/ml with an EE% of 39.5%.

**Table 1:** Physicochemical characterization of the formulations

	Particle size (nm)		Zeta potential (mV)	pH	Span	PDI
	D [3, 4]^a^	*z*-average^b^				
LNC	205 ± 4.4	199 ± 0.2	- 6.40 ± 0.6	5.36 ± 0.3	1.74 ± 0.02	0.10 ± 0.01
Mel-LNC	203 ± 3.6	199 ± 2.5	−5.20 ± 0.2	5.46 ± 0.2	1.77 ± 0.02	0.12 ± 0.04

^a^laser diffratometry; ^b^dynamic light scattering analysis; *LNC* Unloaded lipid-core nanocapsules, *Mel-LNC* Melatonin-loaded lipid-core nanocapsules. Results are expressed as mean ± SD. Number of samples?

### Effects of treatment with LNC, Mel-LNC, and Mel on PQ-induced lipid peroxidation

As according to previous studies by our research group, 30% of the worms exposed to PQ 0.5 mM were not alive 24 h after treatment, in contrast with the control group, where significant mortality was not observed. Pre-treatment with Mel-LNC, before PQ exposure, was capable of significantly increasing the survival rate of the worms (about 25%) when compared with the worms that did not receive the pre-treatment. The same was not observed when the worms were pre-treated with free melatonin.

**Fig. 1:**
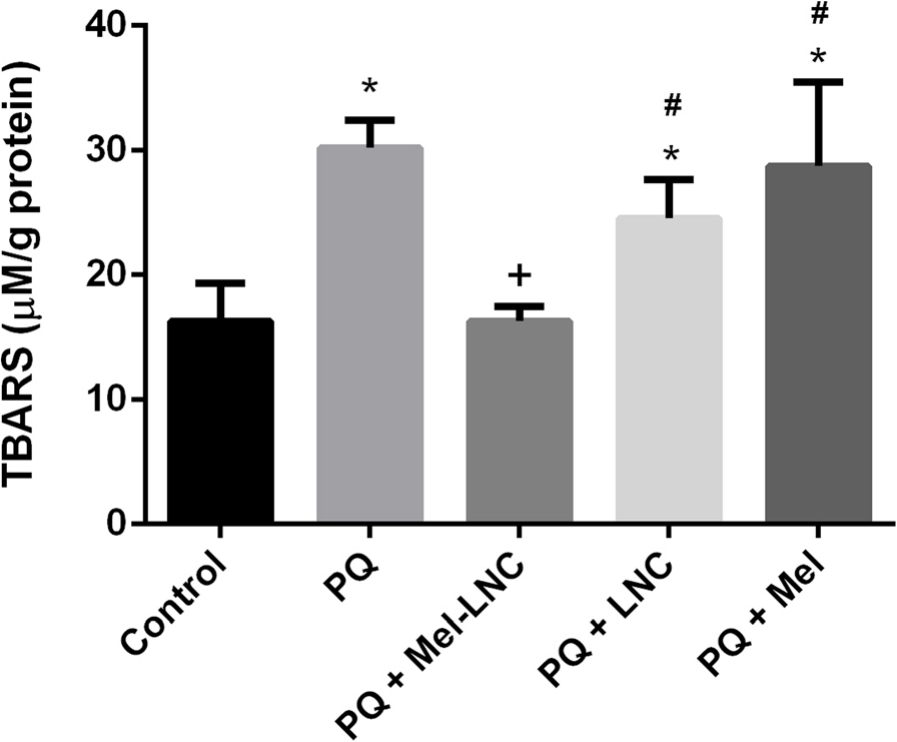
Mel-LNC protects against PQ-induced lipid peroxidation. *p < 0.001 compared to control group; ^+^*p* < 0.001 compared to PQ group; ^#^p < 0.001 compared to PQ + Mel-LNC group

Since lipid peroxidation is one of the toxic effects caused by PQ, we determined the TBARS levels in exposed worms. The lipid peroxidation assay demonstrated a significant increase in TBARS levels in worms exposed to PQ when compared to the control group (Fig. 1; *p* < 0.001). When the worms were pre-treated with free melatonin and LNC, it was not possible to verify any protection from lipid peroxidation inflicted by PQ, whereas Mel-LNC significantly decreased the TBARS levels induced by this herbicide (Fig. 1; p < 0.001).

### Effects of treatment with Mel-LNC and Mel on GFP-tagged protein levels

**Fig. 2:**
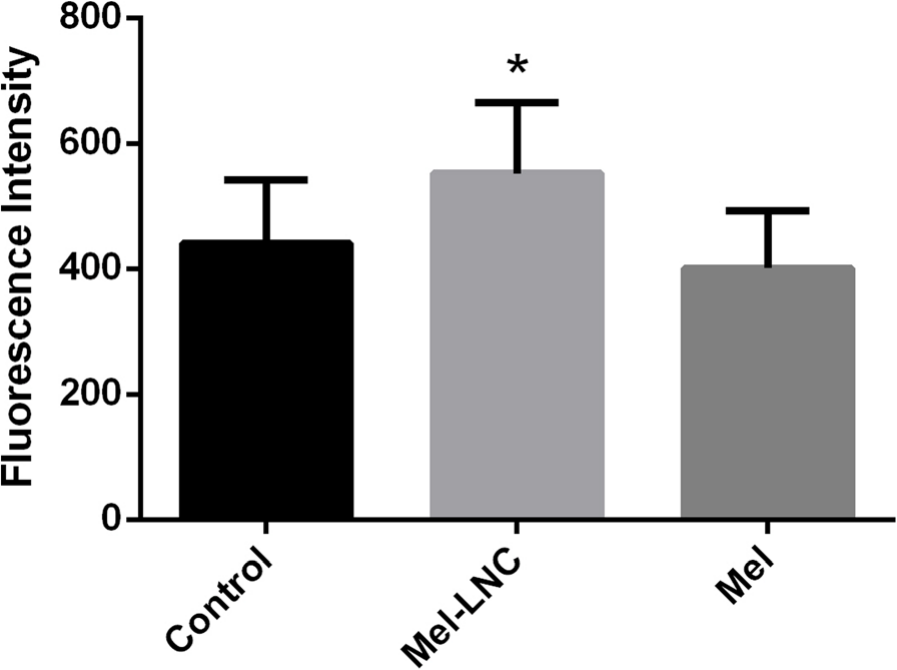
Mel-LNC enhances superoxide dismutase (SOD) activity. *p < 0.05 compared to control group

Regarding the possible mechanism of action of Mel-LNC, it was possible to observe that this formulation was responsible for the increase in fluorescence levels of the transgenic worms CF1553 (Fig. 2). This strain expresses the antioxidant enzyme superoxide dismutase-3 (SOD-3), which is responsible for the detoxification of reactive oxygen species (ROS), reducing the toxic effects induced by overproduction of ROS caused by PQ exposure. These effects were not observed when the worms were exposed to Mel, indicating that free melatonin is less effective in inducing SOD-3 expression, which is responsible for protecting the worms.

## Discussion

In this study, it was demonstrated that Mel-LNC was capable of protecting *C. elegans* from lipid peroxidation caused by PQ. ROS overproduction is the main mechanism of PQ toxicity, inducing cell damage such as membrane injury, leading to the formation of products of lipid peroxidation, measured by TBARS assay. Additionally, Mel-LNC increased the fluorescence intensity of the transgenic strain CF1553 [muls84], which presented homology with antioxidant enzymes SOD-3 in humans. These findings demonstrated that activation of SOD-3 could be involved in mediating Mel-LNC protection from PQ toxicity.

The characterization of the formulations used in this study (LNC and Mel-LNC) presented mean particle sizes similar to other studies that have used nanocapsules prepared by deposition of pre-formed polymer [26, 27]. The characterization of formulations is an essential step in toxicological and pharmacological studies, providing trustworthy results and conclusions. The polymer used in the formulation is responsible for the negative zeta potential values and these values were near zero because of the presence of polysorbate 80. These characteristics make the formulation stable and avoid the formation of aggregates due to a steric repulse mechanism among the particles [27]. The low polydispersity index indicated the narrow particle distribution for both formulations (LNC and Mel-LNC). The total melatonin content in the Mel-LNC was 96.7%, and the EE% was 39.5%, as according to previous studies that have used the same formulation [10, 11]. Furthermore, previous studies have reported that encapsulation of some substances improves their biological action [28–30].

Melatonin and its metabolite are described as potent free radical scavengers [31]. Recently, it was demonstrated that exogenous melatonin reduced oxidative stress induced by hydrogen peroxide (H_2_O_2_) through modulation of the ErK/Akt/NFkB pathway [32]. In addition, Garcia-Rubio et al. [33] showed that melatonin prevented cell damage in hepatocytes caused by PQ in an in vitro assay, confirming that melatonin is responsible for protecting against oxidative stress induced by this herbicide.

The toxic mechanism of PQ is mainly the formation of ROS leading to oxidative stress. PQ is responsible for inhibition of the electron transport chain and reacts with NADPH forming a PQ-radical. This radical reacts with oxygen and generates superoxide radicals. The antioxidant enzyme involved in this detoxification is superoxide dismutase, which converts superoxide anion to a relatively lower active form of hydrogen peroxide [34]. In *C. elegans* it is possible to investigate the involvement of this antioxidant enzyme with strain CF1553. The imbalance between oxidant species and antioxidants results in cell damage, such as lipid peroxidation. Due to its antioxidant capacity, melatonin has been described in cases of acute PQ intoxication [35–37]. In this study, it was possible to observe that Mel-LNC significantly reduced lipid peroxidation in worms exposed to PQ when compared to the PQ, PQ + LNC, and PQ + Mel groups. These findings reinforce one of the advantages of nanotechnology: improving the biological action of substances [38–40]. There are reports describing the biological action of LNC per se [41]; however, in this study, this was not observed.

All cellular components are susceptible to ROS action; however, the lipid membrane is one of the most affected. Higher levels of TBARS were observed in *C. elegans* when exposed to PQ compared to the control group (not exposed to PQ), and this is a good way to evaluate lipid peroxidation in this model [20]. The lipid peroxidation process leads to modifications in the structure and permeability of cell membranes and the formation of secondary products [42].

Melatonin is also described as a modulator of some antioxidant enzymes that help the body to eliminate reactive oxygen and nitrogen species [43]. Our results demonstrated that Mel-LNC significantly increases SOD-3 expression, which is crucial to protect the worms from superoxide radicals formed by PQ. These results were in agreement with a recent study from Choudhary et al. [44], which demonstrated that melatonin was able to reduce lipid peroxidation and increase SOD and CAT activity in ewes. Moreover, the same has been observed in an in vitro assay with fresh hepatocytes, where there was a decrease in MDA levels and modulation of the antioxidant enzymes SOD and CAT in an oxidative stress model induced by H_2_O_2_ [33].

Wen et al. demonstrated that polydatin, a natural resveratrol glycoside, enhanced SOD-3::GFP expression in CF1553 worms, improving oxidative stress resistance in this model [45]. Moreover, an experimental study using two new molecules with antioxidant properties, 4-phenylselanyl- and 4-phenyltellanyl-7-chloroquinoline, showed the involvement of SOD-3 in preventing PQ-induced mortality and lifespan reduction in *C. elegans* [20].

Tambara et al. [46] reported that extract obtained from pitanga fruit (a native Brazilian fruit) presented beneficial effects when *C. elegans* were exposed to stressors (H_2_O_2_ and juglone), and was responsible for activating genes involved in antioxidant defenses, such as SOD-3. It was demonstrated that Se- and Te-xylofuranosides did not present toxic effects in concentrations that prevent and/or reverse the oxidative damage induced by manganese, as well as increasing SOD-3 expression [47].

The better effects of Mel-LNC observed in this study could be due to the mechanism of distribution of melatonin when this molecule is associated with polymeric nanocapsules and the characteristics of LNC per se. About 30–40% of melatonin is incorporated into the nanocapsules and this leads to a large load arrival of this molecule in cells, in this case in lipid membranes, where each nanocapsule present in the suspension can carry about 1200 molecules of melatonin. To our knowledge, this is the first time that the involvement of nanoencapsulated melatonin in SOD-3 expression has been demonstrated in *C. elegans*. These findings show that the protective activity against oxidative stress induced by PQ is closely associated with the increase of antioxidant enzyme SOD-3 by Mel-LNC in *C. elegans*.

## Conclusion

Taken together, these results show that pre-treatment with MEL-LNC decreased lipid peroxidation probably through modulation of SOD enzymatic activity. Interestingly, these results were only observed in *C. elegans* treated with nanoencapsuled melatonin. Therefore, in this study Mel-LNC was more effective against paraquat damage, showing that nanotechnology presents a useful tool for improving the characteristics and biological properties of melatonin.

## Data Availability

The data for this study are available from the corresponding author on reasonable request.

## Abbreviations

EE%: Encapsulation efficiency
H_2_O_2_: Hydrogen peroxide
LNC: Unloaded lipid-core nanocapsules
LOO: Lipid peroxyl readical
MDA: Malondialdehyde
Mel: Free melatonin
Mel-LNC: Melatonin-loaded lipid-core nanocapsules
NADPH: Nicotinamide adenine dinucleotide phosphate
NGM: Nematode growth medium
OS: Oxidative stress
PCL: Poly(ɛ-caprolactone)
PQ: Paraquat
ROS: Reactive oxygen species
SOD-3: Superoxide dismutase-3
TBA: Thiobarbituric acid
TBARS: Thiobarbituric acid reactive substances
